# Supplementary material for: Use of isotretinoin among girls and women of childbearing age and occurrence of isotretinoin-exposed pregnancies in Germany: A population-based study
Source: PLoS Med. 2024 Jan 25;21(1):e1004339. doi: 10.1371/journal.pmed.1004339 (PMC10810459; doi:10.1371/journal.pmed.1004339)
Supplement: S1 Checklist — (DOCX) [file pmed.1004339.s001.docx]

**S1 Checklist: STROBE Statement—checklist of items that should be included in reports of observational studies**

|  | Item No | Recommendation | Section & Paragraph |
| --- | --- | --- | --- |
| **Title and abstract** | 1 | (*a*) Indicate the study’s design with a commonly used term in the title or the abstract | Use of isotretinoin among girls and women of childbearing age and occurrence of isotretinoin-exposed pregnancies in Germany: **A descriptive study** |
|  |  | (*b*) Provide in the abstract an informative and balanced summary of what was done and what was found | See Abstract |
| Introduction | | | |
| Background/rationale | 2 | Explain the scientific background and rationale for the investigation being reported | Introduction, paragraphs #1-3 |
| Objectives | 3 | State specific objectives, including any prespecified hypotheses | Introduction, paragraph #4 |
| Methods | | | |
| Study design | 4 | Present key elements of study design early in the paper | Methods, sections “Study design and study population”, “Identification of exposed pregnancies”, “Exploration of potential malformations among exposed children” |
| Setting | 5 | Describe the setting, locations, and relevant dates, including periods of recruitment, exposure, follow-up, and data collection | Methods, sections “Data Source”, “Study design and study population”, “Identification of exposed pregnancies”, “Exploration of potential malformations among exposed children” |
| Participants | 6 | (*a*) *Cohort study*—Give the eligibility criteria, and the sources and methods of selection of participants. Describe methods of follow-up  *Case-control study*—Give the eligibility criteria, and the sources and methods of case ascertainment and control selection. Give the rationale for the choice of cases and controls  *Cross-sectional study*—Give the eligibility criteria, and the sources and methods of selection of participants | Methods, sections “Study design and study population”, “Identification of exposed pregnancies”, “Exploration of potential malformations among exposed children” |
|  |  | (*b*) *Cohort study*—For matched studies, give matching criteria and number of exposed and unexposed  *Case-control study*—For matched studies, give matching criteria and the number of controls per case | n.A., as no matching was done |
| Variables | 7 | Clearly define all outcomes, exposures, predictors, potential confounders, and effect modifiers. Give diagnostic criteria, if applicable | Methods, sections “Study design and study population”, “Identification of exposed pregnancies”, “Exploration of potential malformations among exposed children” |
| Data sources/ measurement | 8* | For each variable of interest, give sources of data and details of methods of assessment (measurement). Describe comparability of assessment methods if there is more than one group | Methods, sections “Data Source”, “Study design and study population”, “Identification of exposed pregnancies”, “Exploration of potential malformations among exposed children” |
| Bias | 9 | Describe any efforts to address potential sources of bias | Methods, sections “Study design and study population”, “Identification of exposed pregnancies”, “Exploration of potential malformations among exposed children” |
| Study size | 10 | Explain how the study size was arrived at | Methods, sections “Study design and study population”, “Identification of exposed pregnancies”, “Exploration of potential malformations among exposed children” |
| Quantitative variables | 11 | Explain how quantitative variables were handled in the analyses. If applicable, describe which groupings were chosen and why | Methods, section “Data analysis” |
| Statistical methods | 12 | (*a*) Describe all statistical methods, including those used to control for confounding | Methods, section “Data analysis” |
|  |  | (*b*) Describe any methods used to examine subgroups and interactions | Methods, section “Data analysis” |
|  |  | (*c*) Explain how missing data were addressed | n.A. no missing data due to the study being based on claims data |
|  |  | (*d*) *Cohort study*—If applicable, explain how loss to follow-up was addressed  *Case-control study*—If applicable, explain how matching of cases and controls was addressed  *Cross-sectional study*—If applicable, describe analytical methods taking account of sampling strategy | Cross-sectional study: Methods, sections “Study design and study population”, “Data analysis” |
|  |  | (*e*) Describe any sensitivity analyses | Methods, section “Identification of exposed pregnancies” |

| Results | | | |
| --- | --- | --- | --- |
| Participants | 13* | (a) Report numbers of individuals at each stage of study—e.g., numbers potentially eligible, examined for eligibility, confirmed eligible, included in the study, completing follow-up, and analysed | n.A. |
|  |  | (b) Give reasons for non-participation at each stage | n.A. |
|  |  | (c) Consider use of a flow diagram | n.A. |
| Descriptive data | 14* | (a) Give characteristics of study participants (eg demographic, clinical, social) and information on exposures and potential confounders | Table 1 |
|  |  | (b) Indicate number of participants with missing data for each variable of interest | n.A. no missing data due to the study being based on claims data |
|  |  | (c) *Cohort study*—Summarise follow-up time (eg, average and total amount) | n.A. |
| Outcome data | 15* | *Cohort study*—Report numbers of outcome events or summary measures over time | n.A. |
|  |  | *Case-control study—*Report numbers in each exposure category, or summary measures of exposure | n.A. |
|  |  | *Cross-sectional study—*Report numbers of outcome events or summary measures | Results, sections “Prevalent use of isotretinoin among girls and women of childbearing age” “Characterization of exposed pregnancies”, “Characterization of exposed children” |
| Main results | 16 | (*a*) Give unadjusted estimates and, if applicable, confounder-adjusted estimates and their precision (eg, 95% confidence interval). Make clear which confounders were adjusted for and why they were included | n.A. descriptive analysis, no adjustments performed |
|  |  | (*b*) Report category boundaries when continuous variables were categorized | n.A. no categorization of continuous variables performed |
|  |  | (*c*) If relevant, consider translating estimates of relative risk into absolute risk for a meaningful time period | n.A. no measures of relative risk assessed |
| Other analyses | 17 | Report other analyses done—e.g., analyses of subgroups and interactions, and sensitivity analyses | Results, sections “Characterization of exposed pregnancies” paragraph #2, “Characterization of exposed children” |
| Discussion | | | |
| Key results | 18 | Summarise key results with reference to study objectives | Discussion, paragraph #1 |
| Limitations | 19 | Discuss limitations of the study, taking into account sources of potential bias or imprecision. Discuss both direction and magnitude of any potential bias | Discussion section paragraph #7 |
| Interpretation | 20 | Give a cautious overall interpretation of results considering objectives, limitations, multiplicity of analyses, results from similar studies, and other relevant evidence | Discussion section, paragraph #2, paragraph #3 |
| Generalisability | 21 | Discuss the generalisability (external validity) of the study results | Discussion section, paragraph #2, paragraph #3 |
| Other information | | | |
| Funding | 22 | Give the source of funding and the role of the funders for the present study and, if applicable, for the original study on which the present article is based | Submission form metadata |

*Give information separately for cases and controls in case-control studies and, if applicable, for exposed and unexposed groups in cohort and cross-sectional studies.

**Note:** An Explanation and Elaboration article discusses each checklist item and gives methodological background and published examples of transparent reporting. The STROBE checklist is best used in conjunction with this article (freely available on the Web sites of PLoS Medicine at http://www.plosmedicine.org/, Annals of Internal Medicine at http://www.annals.org/, and Epidemiology at http://www.epidem.com/). Information on the STROBE Initiative is available at www.strobe-statement.org.
