# Supplementary material for: Use of isotretinoin among girls and women of childbearing age and occurrence of isotretinoin-exposed pregnancies in Germany: A population-based study
Source: PLoS Med. 2024 Jan 25;21(1):e1004339. doi: 10.1371/journal.pmed.1004339 (PMC10810459; doi:10.1371/journal.pmed.1004339)
Supplement: S1 Table — (DOCX) [file pmed.1004339.s002.docx]

**Appendix**

**S1 Table: Number of girls and women aged 13–49 years with at least one dispensation of isotretinoin between 2004 and 2019 in GePaRD, by age group and year of prescription**

| **Age group** | **Study year** | | | | | | | | | | | | | | | | |
| --- | --- | --- | --- | --- | --- | --- | --- | --- | --- | --- | --- | --- | --- | --- | --- | --- | --- |
|  | **2004** (n=3,798) | **2005** (n=3,431) | **2006** (n=3,737) | **2007** (n=4,238) | **2008** (n=4,456) | **2009** (n=4,907) | **2010** (n=5,331) | **2011** (n=5,319) | **2012** (n=5,741) | **2013** (n=6,531) | **2014** (n=6,961) | **2015** (n=7,071) | **2016** (n=7,377) | **2017** (n=7,660) | **2018** (n=7,504) | **2019** (n=7,663) | **Total study population*** |
| 13–15 years | 232  (6.1%) | 194  (5.7%) | 214  (5.7%) | 242  (5.7%) | 284  (6.4%) | 330  (6.7%) | 358  (6.7%) | 370 (7.0%) | 405 (7.1%) | 479 (7.3%) | 468 (6.7%) | 456 (6.4%) | 397 (5.4%) | 406 (5.3%) | 381 (5.1%) | 378 (4.9%) | 50,936 |
| 16–20 years | 697 (18.4%) | 617 (18.0%) | 699 (18.7%) | 780 (18.4%) | 877 (19.7%) | 898 (18.3%) | 927 (17.4%) | 1,019 (19.2%) | 1,090 (19.0%) | 1,318 (20.2%) | 1,484 (21.3%) | 1,582 (22.4%) | 1,673 (22.7%) | 1,751 (22.9%) | 1,690 (22.5%) | 1,644 (21.5%) |  |
| 21–25 years | 737 (19.4%) | 604 (17.6%) | 700 (18.7%) | 840 (19.8%) | 848 (19.0%) | 960 (19.6%) | 1,101 (20.7%) | 1,030 (19.4%) | 1,089 (19.0%) | 1,368 (20.9%) | 1,430 (20.5%) | 1,499 (21.2%) | 1,614 (21.9%) | 1,759 (23.0%) | 1,875 (25.0%) | 1,917 (25.0%) |  |
| 26–30 years | 552 (14.5%) | 545 (15.9%) | 595 (15.9%) | 682 (16.1%) | 704 (15.8%) | 841 (17.1%) | 975 (18.3%) | 921 (17.3%) | 1,006 (17.5%) | 1,085 (16.6%) | 1,237 (17.8%) | 1,245 (17.6%) | 1,346 (18.2%) | 1,497 (19.5%) | 1,425 (19.0%) | 1,442 (18.8%) |  |
| 31–35 years | 444 (11.7%) | 391 (11.4%) | 403 (10.8%) | 410  (9.7%) | 425  (9.5%) | 504 (10.3%) | 546 (10.2%) | 610 (11.5%) | 673 (11.7%) | 736 (11.3%) | 728 (10.5%) | 757 (10.7%) | 775 (10.5%) | 771 (10.1%) | 798 (10.6%) | 802 (10.5%) |  |
| 36–40 years | 551 (14.5%) | 472 (13.8%) | 482 (12.9%) | 527 (12.4%) | 491 (11.0%) | 480 ‘(9.8%) | 469  (8.8%) | 450 (8.5%) | 495 (8.6%) | 534 (8.2%) | 535 (7.7%) | 542 (7.7%) | 598 (8.1%) | 588 (7.7%) | 554 (7.4%) | 665 (8.7%) |  |
| 41–45 years | 400 (10.5%) | 411 (12.0%) | 432 (11.6%) | 491 (11.6%) | 532 (11.9%) | 549 (11.2%) | 557 (10.4%) | 524 (9.9%) | 577 (10.1%) | 566 (8.7%) | 596 (8.6%) | 523 (7.4%) | 496 (6.7%) | 481 (6.3%) | 439 (5.9%) | 456 (6.0%) |  |
| 46–49 years | 185  (4.9%) | 197  (5.7%) | 212  (5.7%) | 266  (6.3%) | 295  (6.6%) | 345  (7.0%) | 398  (7.5%) | 395 (7.4%) | 406 (7.1%) | 445 (6.8%) | 483 (6.9%) | 467 (6.6%) | 478 (6.5%) | 407 (5.3%) | 342 (4.6%) | 359 (4.7%) |  |
| * Total number of girls / women aged 13–49 years with at least one dispensation of isotretinoin during the study period.  Note: This number is not the sum of the total number of each year given that a woman can have a dispensation of isotretinoin in different years of the study period and is then counted in each of these years but only once for the total sum. | | | | | | | | | | | | | | | | | |
