# Supplementary material for: Use of isotretinoin among girls and women of childbearing age and occurrence of isotretinoin-exposed pregnancies in Germany: A population-based study
Source: PLoS Med. 2024 Jan 25;21(1):e1004339. doi: 10.1371/journal.pmed.1004339 (PMC10810459; doi:10.1371/journal.pmed.1004339)
Supplement: S2 Table — (DOCX) [file pmed.1004339.s003.docx]

**S2** **Table: Prescriptions of Isotretinoin dispensed to girls and women aged 13–49 years between 2004 and 2019 in GePaRD: Distribution of the specialty of the prescribing physician**

| **Specialty of the prescribing physician** | **Number of dispensations of isotretinoin (N=339,408)** |
| --- | --- |
| Dermatologist | 302,162 (89.0%) |
| General practitioner | 20,446 (6.0%) |
| Specialist for internal Medicine | 4,038 (1.2%) |
| Gynecologist | 1,296 (0.4%) |
| Other specialist | 6,596 (1.9%) |
| Assessment of specialty not possible | 4,870 (1.4%) |
