# Supplementary material for: Use of isotretinoin among girls and women of childbearing age and occurrence of isotretinoin-exposed pregnancies in Germany: A population-based study
Source: PLoS Med. 2024 Jan 25;21(1):e1004339. doi: 10.1371/journal.pmed.1004339 (PMC10810459; doi:10.1371/journal.pmed.1004339)
Supplement: S3 Table — (DOCX) [file pmed.1004339.s004.docx]

**S3 Table: Number of pregnancies exposed to isotretinoin between 2004 and 2019 in GePaRD by age group and year of beginning of pregnancy**

| **Age group** | **Study year** | | | | | | | | | | | | | | | | |
| --- | --- | --- | --- | --- | --- | --- | --- | --- | --- | --- | --- | --- | --- | --- | --- | --- | --- |
|  | **2004** | **2005** | **2006** | **2007** | **2008** | **2009** | **2010** | **2011** | **2012** | **2013** | **2014** | **2015** | **2016** | **2017** | **2018** | **2019** | **Total** |
| 13-15 years | 0  (0.0%) | 0  (0.0%) | 0  (0.0%) | 0  (0.0%) | 0  (0.0%) | 0  (0.0%) | 0  (0.0%) | 0  (0.0%) | 0  (0.0%) | 0  (0.0%) | 0  (0.0%) | 0  (0.0%) | 1  (7.1%) | 0  (0.0%) | 0  (0.0%) | 0  (0.0%) | 1  (0.6%) |
| 16-20 years | 0  (0.0%) | 2  (40.0%) | 2  (18.2%) | 2  (18.2%) | 1  (25%) | 1  (12.5%) | 1  (11.1%) | 1  (16.7%) | 2  (16.7%) | 4  (20.0%) | 0  (0.0%) | 0  (0.0%) | 2  (14.3%) | 2  (16.7%) | 1  (4.8%) | 0  (0.0%) | 21  (11.8%) |
| 21-25 years | 1  (33.3%) | 2  (40.0%) | 7  (63.6%) | 1  (9.1%) | 0  (0.0%) | 4  (50.0%) | 2  (22.2%) | 2  (33.3%) | 3  (25%) | 3  (15%) | 1  (12.5%) | 2  (15.4%) | 2  (14.3%) | 1  (8.3%) | 5  (23.8%) | 9  (42.9%) | 45  (25.3%) |
| 26-30 years | 1  (33.3%) | 0  (0.0%) | 1  (9.1%) | 3  (27.3%) | 1  (25%) | 0  (0.0%) | 1  (11.1%) | 2  (33.3%) | 4  (33.3%) | 5  (25%) | 2  (25%) | 4  (30.8%) | 4  (28.6%) | 3  (25%) | 10  (47.6%) | 5  (23.8%) | 46  (25.8%) |
| 31-35 years | 1  (33.3%) | 0 (0.0%) | 0  (0.0%) | 5  (45.5%) | 1  (25%) | 2  (25%) | 3  (33.3%) | 0  (0.0%) | 1  (8.3%) | 6  (30.0%) | 2  (25%) | 5  (38.5%) | 4  (28.6%) | 4  (33.3%) | 4  (19%) | 6  (28.6%) | 44 (24.7%) |
| 36-40 years | 0  (0.0%) | 1  (20.0%) | 1  (9.1%) | 0  (0.0%) | 1  (25%) | 1  (12.5%) | 2  (22.2%) | 1  (16.7%) | 2  (16.7%) | 1  (5%) | 2  (25%) | 2  (15.4%) | 1  (7.1%) | 2  (16.7%) | 1  (4.8%) | 1  (4.8%) | 19  (10.7%) |
| 41-45 years | 0  (0.0%) | 0  (0.0%) | 0  (0.0%) | 0  (0.0%) | 0  (0.0%) | 0  (0.0%) | 0  (0.0%) | 0  (0.0%) | 0  (0.0%) | 1  (5%) | 1  (12.5%) | 0  (0.0%) | 0  (0.0%) | 0  (0.0%) | 0  (0.0%) | 0  (0.0%) | 2  (1.1%) |
| 46-49 years | 0  (0.0%) | 0  (0.0%) | 0  (0.0%) | 0  (0.0%) | 0  (0.0%) | 0  (0.0%) | 0  (0.0%) | 0  (0.0%) | 0  (0.0%) | 0  (0.0%) | 0  (0.0%) | 0  (0.0%) | 0  (0.0%) | 0  (0.0%) | 0  (0.0%) | 0  (0.0%) | 0  (0.0%) |
| Total | 3 | 5 | 11 | 11 | 4 | 8 | 9 | 6 | 12 | 20 | 8 | 13 | 14 | 12 | 21 | 21 | 178 |
